# A temperature-dependent phenology model for the greenhouse whitefly *Trialeurodes vaporariorum* (Hemiptera: Aleyrodidae)

**DOI:** 10.1016/j.virusres.2020.198107

**Published:** 2020-11

**Authors:** Heidy Gamarra, Marc Sporleder, Pablo Carhuapoma, Jürgen Kroschel, Jan Kreuze

**Affiliations:** Crop Systems Intensification and Climate Change, International Potato Center (CIP), Av. La Molina 1895, Lima 12, Peru International Potato Center (CIP), Apartado 1558, Lima 12, Peru

**Keywords:** Whiteflies, Modeling, Phenology, Temperature-dependent development, Development rate models, Life-table statistics, Potato pests, Virus transmission, Virus vectors

## Abstract

The greenhouse whitefly *Trialeurodes vaporariorum* Westwood (Hemiptera: Aleyrodidae) is a serious pest of many fruits, vegetables and ornamental crops in sub-tropical regions and in greenhouses worldwide. Potato is a secondary host of the species but the major threat from this insect in the Andean region for potato is the transmission by this insect of a crinivirus causing Potato yellow vein disease. Determination of the pest’s temperature-dependent population growth potential is crucial knowledge for understanding the population dynamics and spread potential of the species and the diseases it can transmit, as well as for designing effective pest management strategies. *Trialeurodes vaporariorum* development, mortality and reproduction were studied at seven constant temperatures ranging from 10 to 32 °C. The Insect Life Cycle Modeling (ILCYM) software was used to fit nonlinear equations to the data and establish an overall phenology model to simulate life-table parameters based on temperature. In addition, life tables of *T. vaporariorium* were established at daily fluctuating temperature in two different environments: Cusco (5 °C–35 °C) and La Molina, Lima (13.7 °C–24.9 °C), and used to adjust and validate the model. The insect only completed its life cycle at constant temperatures above 15 °C and below 32 °C although the cycle was completed at daily fluctuating temperatures between 5 °C and 35 °C. The overall model portrayed population development within the temperature range of 14° to 32 °C with a maximum finite rate of population increase (= 1.14) at 23 °C. However, the model revealed poor convergence with life tables established at fluctuating temperatures indicating an influential effect of temperature fluctuations on the whitefly life history parameters, particularly on adult survival time and reproduction. Therefore, we adjusted the model for convergence with a single life table observed at fluctuating temperature. The adjusted model gave good predictions when compared with remaining observed life tables and published data. The adjusted model predicted population development within the temperature range of 11.5 °C–35.5 °C, and maximum population growth at around 24 °C with a finite rate of increase, *λ*, of 1.137 and a population doubling time of 5 days. The established process-based physiological model presented here for *T. vaporariorum* can be used for predicting the species distribution potential based on temperature worldwide and should prove helpful in adjusting pest management measures. Moreover, the information obtained will be used to predict the spread potential of potato yellow vein disease.

## Highlights

• Development, mortality and reproduction of *T. vaporariorum* were studied at constant temperatures ranging from 10 to 32 °C.
• Nonlinear equations were fitted to the data and a temperature-driven process-based phenology/population growth model for the vector pest established.
• After adjustment, the model gave good predictions when compared with observed life tables and published data.
• The model can be used for predicting the species distribution potential based on temperature worldwide and adjusting pest management measures.

## 1 Introduction

The greenhouse whitefly *Trialeurodes vaporariorum* Westwood (Hemiptera: Aleyrodidae) is a primary insect pest of many fruit, vegetable and ornamental crops throughout the world. The species has become invasive and is today reported in more than 71 countries (EPPO, 2019; Evans, 2007; Byrne et al., 1990). The center of origin for this pest has not been identified. According to Vet et al. (1980), the species is most probably indigenous to tropical and subtropical America. In Europe and other temperate regions, *T. vaporariorum* is frequently observed in greenhouses and other protected horticultural environments (polytunnels) (EPPO, 2020). In South America, the whitefly has long been recorded on annual field crops in highland areas of Colombia (Guzman-Barney et al., 2012), Ecuador (Anderson and Morales, 2005) and Peru (Valencia, 2000). The species is confirmed as a vector of numerous plant viruses (Diaz and Pulgarin, 1989; Wintermantel, 2004., Wisler et al., 1998; Salazar et al., 2000; Tzanetakis et al., 2006).

In the early 1980’s, *T. vaporariorum* became a serious pest of several crops in the Andes. Major outbreaks occurred in 1987, 1991 and 1994 in selected areas of Colombia, northern Ecuador and the Constanza Valley in Dominican Republic (Alvarez and Abud-Atún, 1995). At the end of 1996, *T. vaporariorum* was a predominant species of industrial tomatoes in the Valley of Ica, Peru, where it became a key pest (Valencia, 2000) but was later displaced by the more aggressive whitefly *Bemisia tabaci* (Gennadius); however, in Peru and several other South American countries, *T. vaporariorum* remains a significant threat to potato production due to its transmission of the potato yellow vein virus (PYVV, genus *Crinivirus*, family *Closteroviridae*) that causes potato yellow vein disease, which significantly reduces potato yields (Salazar et al., 2000). Guzman-Barney et al. (2012) reported yield reductions of between 25 and 50 % in Colombia, while Salazar et al. (2000) observed up to 80 % yield losses in Peru for the Canchan cultivar (INIA: CIP380389.1) due to the disease.

Temperature is a critical abiotic factor affecting the development, survival, and reproduction of insect species. The ability of an insect to develop at different temperatures is an important adaptation to survive in various climatic conditions, and its understanding is important for predicting pest outbreaks (Gilbert and Raworth, 1996; Mujica et al., 2017). One of the main factors that influence the development of larger populations of whiteflies in agricultural regions of Latin America is the diversification of crops which provide increased availability of hosts for whiteflies and has also contributed to a significant increase in the use of agrochemicals (Anderson and Morales, 2005). In addition, climate change (drought and heat) and more international exchange of plant material have facilitated the development and dissemination of the pest. Although the biology and ecology of *T. vaporariorum* has been extensively studied in different parts of the world under laboratory condition (Manzano and Lenteren, 2009), few degree-day models for specific potato regions exist (González-Dufau et al., 2018).

Insect life-table data obtained under a wide range of different temperatures provide the information for developing temperature-based phenology models, which are helpful for understanding pest population growth potentials in different agroecologies (Briere et al., 1999; Kroschel et al., 2012; Sporleder et al., 2004, 2016). Degree-day models, which have been established for describing development in many insect pests, employ linear models using accumulation of temperature above the minimum temperature threshold (Nietschke et al., 2007). However, due to the non-linearity of the development curve, especially when temperature deviates from the intrinsic optimal temperature of the species, these models become poor predictors of insect development. This method works well for intermediate temperatures but produces errors (significant deviations from the real development) when daily temperature fluctuates to extremes (Stinner et al., 1974; Worner, 1992). More progressive models use nonlinear models of higher biological significance (i.e., Logan et al., 1976; Sharpe and DeMichele, 1977), and include stochastic functions for variability in development times within a population among individuals (Sharpe et al., 1981; Wagner et al., 1984).

Knowledge of the insect pest phenology and population biology is elemental for designing effective pest and disease management measures. The present study is part of the effort to establish phenology models for major insect pests of potato and map the establishment risk and performance capacity of these pests in potato production regions worldwide (Kroschel et al., 2016). The objective of this study was to establish a temperature-driven phenology model for *T. vaporariorum* to enable simulation of population development of the whitefly as an indicator for risk of virus spread. Life table data were collected at different constant temperatures under controlled conditions and the data used to determine the nonlinear relationship between temperature and the whitefly’s development, mortality, and reproduction. The established functions were used to compile a process-based temperature-driven overall phenology model for the pest that allowed predicting life table parameters for the species based on temperature. The simulation model results were verified with life tables established under naturally fluctuating temperatures. Model building and simulations were made using the Insect Life Cycle Modeling (ILCYM) software developed by the International Potato Center, Lima, Peru (Sporleder et al., 2013, 2017). Although a lot of research has been addressing the effects of temperature on *T. vaporariorum*, no model has been developed to simulate the full life history of the species in potato.

## 2 Materials and methods

### 2.1 Insect rearing

A colony of *T. vaporariorum* was initiated with puparia collected from *Lantana* sp. (Verbenaceae) plantings in La Molina, Lima, Peru. The colony was maintained and mass-reared in the greenhouse at 18−23 °C, 80 % RH and a photoperiod of 12:12 h light (L): dark (D). For this, the petioles of the whitefly-infected Lantana leaves collected from the field were placed on a sponge soaked in water on a tray to ensure that they were in contact with the water until the emergence of adult whiteflies. Adults were maintained on potato (cv. Canchan) in insect-proof cages, which contained two sleeves for in-situ manipulation. For providing oviposition medium, four new potato plants were placed inside the cage and replaced after a period of five days. Since the Lantana leaves were partly co-infested with *B. tabaci*, after a period of 25 days the infested plants were moved to new cages; with a style all puparia of *B. tabaci* and parasitized puparia of *T. vaporariorum* were eliminated leaving only healthy puparia of *T. vaporariorum*. After rearing the third whitefly generation on potato, collected adults were used to initiate temperature experiments.

### 2.2 Experimental procedures for data collection

The effects of temperature on the biology of *T. vaporariorum* were studied on life tables initiated with a number of 100 new-laid eggs and incubated in controlled incubation chambers at seven constant temperatures of 10°, 15°, 18°, 20°, 25°, 30°, and 32 °C (Table 1). The temperature and relative humidity inside the incubation chambers were monitored using indoor data loggers (Hobo H8, Onset, MA). Relative humidity in the chambers was maintained above 80 % and the photoperiod regime was kept at 12:12 h light (L): dark (D).

**Table 1:** Median development times resulting from accelerated failure time modeling and observed survival rates in the immature *T. vaporariorum* life stages at constant temperatures.

Temp.		Eggs			Nymphs			Pupae		
(°C)	N^A^	Median dev. time (days)^C^		Mortality (%)^D^	Median dev. time (days)		Mortality (%)	Median dev. time (days)		Mortality (%)
10	100	33.1 (28.8-37.4)a		87 (±3.3)	-		100 (±0)	n.a.		
15	100	17.3 (14.9-19.7)b		0	17.5 (16.4-18.7)a		14 (±3.5)	11.6 (9.4-13.8)a		24 (±4.6)
18	100	9.6 (8.3-11)c		0	13.8 (12.1-15.5)b		13 (±3.4)	6.1 (4.6-7.7)b		13 (±3.6)
20	100	9 (7.7-10.3)c		0	11.2 (10-12.4)bc		4 (±2)	4.9 (3.7-6.1)bc		5 (±2.3)
25	100	5.2 (4.4-5.9)d		0	10.4 (9.2-11.5)c		1 (±1)	3.4 (2.6-4.3)c		10 (±3)
28	100	5 (4.3-5.7)d		0	10.2 (9-11.3)c		14 (±3.4)	6.8 (4.9-8.6)b		30 (±5)
32	100	6.3 (5.4-7.2)d		24 (±4.3)	-		100 (±0)	n.a.		
Model^B^		log-logistic			log-logistic			Weibull		
*ln*(scale)		−3.121 (0.043)***			−2.626 (0.041)***			−1.212 (0.041)***		
Scale δ		0.044 (0.002)***			0.072 (0.003)***			0.298 (0.012)***		
α = 1/ δ		22.7 (0.97)***			13.8 (0.56)***			3.4 (0.14)***		
		Likelihood ratio test			Likelihood ratio test			Likelihood ratio test		
		*ln* L	ΔDeviance	*df*	*ln* L	ΔDeviance	*df*	*ln* L	ΔDeviance	*df*

Intercept only		−1670.3	2184.2	29	−1154.6	835.5	38	−996.5	631.6	40
l for each Temp.		−683.8	211.2	23	−863.8	253.9	34	−792.6	223.8	36
Saturated model		−578.2		(n=31)	−736.8		(n=40)	−680.7		(n=42)
*F*(*df*_x_,*df*_x-1_)		35.8			19.5			16.4		
P		<0.001			0.005			0.007		

A N is the number of individuals evaluated at a given temperature.

B *δ* is the scale of the selected distribution link function; the figures in () are SE of *ln*(*δ*), *δ*, and α (“***” indicates P < 0.001). The accumulated development frequency in relation to normalized age (time/median time) is calculated according to the selected distribution link function; for example, for the log-logistic link function: accu. dev. freq. = 1-(1/(1+*x^α^*)), where *x* is the normalized age (determined through rate summation), and *α* = 1./*δ*.

C Numbers in parenthesis are 95 % confidence limits based on *t*-distribution (a heterogeneity factor, *H* = *deviance*/*df*, was included to calculated the limits). Medians followed by different letters in the same columns are significantly different (P < 0.05) according to the AFT model.

D Numbers in parenthesis are SE calculated using the formula: SE = sqrt([m*{1-*m*}]/*N*), where m is the mortality rate and *N* is the number of test insects.

Each life table was established as follows:

Five 15-day old vigorous and uniformly developed potted 4-leaved potato seedlings of the variety Canchan (INIA: CIP380389.1) were covered individually with a 1 L plastic cup (∅ 12 cm). The plastic cups were modified cutting a hole (∅ 6.5 cm) into the lower part, which was replaced with fine muslin (0.1 μm) for ventilation. To initiate life table temperature experiments, whitefly adults were collected from the insect rearing using an insect aspirator (Bio Quip Products, CA). After aspirating the required number of adults (50 adults per plant), the adults were transferred to a 4-leaf potato plant in a plant pot sealed with muslin (henceforth referred to as mini-cage) and then incubated for 24-h period in a temperature-controlled chamber at the required constant temperature to allow adult females to lay eggs onto the plants. After the 24 h-oviposition period, all flies were removed from the mini-cages using an aspirator (Bio Quip Products, CA). Oviposited *T. vaporariorum* eggs on the plants were identified with the help of a stereoscope and the location of each egg on the potato leaf marked using an indelible marker until a number of 100 eggs, used for initiating one life table experiment, were reached. Excess eggs on the plants were removed using a style; eggs were marked or removed so that each of the 4 leaves per plant contained 5 eggs, thus each of the five plants per temperature contained always 20 eggs. The mini-cages were labeled, sealed, and incubated in a growth chamber at the required temperature.

The development and survival of each test insect were observed daily during the egg stage, and nymph instars until the test insect reached puparium (also referred to as the fourth nymphal instar). During the daily evaluation process, newly developed puparia were transferred individually to small petri-dishes that remained in the incubation chamber of the required temperature and were evaluated daily until adult emergence. Emerging adults were sexed and released individually into a mini-cage, which was incubated at the required temperature, for assessing adult survival time and daily fecundity of females. For evaluating daily fecundity, the female was transferred to a new mini-cage during the evaluation process for easier assessment of the number of eggs oviposited during the past 24-h period; eggs on the plant were recognized using a stereoscope and marked to differentiate them from the new eggs laid the next day. Thereafter, the female was retransferred to the original mini-cage and returned to the incubation chamber. This evaluation was repeated daily until all females died.

### 2.3 Data for model validation

Life tables of *T. vaporariorum* were established at naturally fluctuating temperatures in two locations, *a)* the experimental station of CIP in La Molina–Lima (12° 05′ S, 76° 57′ W, 250 m.a.s.l.) from September to November 2017 (one life table) and the Experimental Station Andenes of the National Institute of Agrarian Innovation (INIA), located in the in the southern highlands of Peru (Zurite district, Anta province, Cusco; 13° 25′ S, 72° 18′ W, 3392 m.a.s.l.) from May to December 2010 (3 temporally consecutive life tables, offspring from the previous life table was used in the following life table). The life tables were established following same methods as used in the constant temperature experiments. A data logger (HOBO, Onset, MA) was located at each experimental site close to the mini-cages in the field for monitoring temperature and relative humidity in 1-h intervals throughout the course of experiments.

### 2.4 Data analysis and modeling

The data were analyzed and the phenology model developed using the Insect Life Cycle Modeling (ILCYM) software Version 4.0 (developed by CIP). The ILCYM software uses R-statistics (R Core Team, 2018) for all statistical calculations and is freely available from the institute’s website https://cipotato.org/riskatlasforafrica/ilcym/ (Sporleder et al., 2017). Data collected (life tables established at the seven constant temperatures for building the model and the four life tables established at fluctuating temperature for validating the model) are available as complementary material on the same webpage. Since the statistical methods used for model building and validation are described elsewhere (ILCYM manual for Version 4, Sporleder et al., 2017) these are described here in brief only.

The life table data were transformed into interval censored time-to-event data (the events occurred between two observation times) in ILCYM. The data on development time of the different immature life stages, adult longevity, and fecundity of females were submitted to survival analysis. Because the data are interval censored medians cannot be assessed correctly. Parametric accelerated failure time (AFT) modeling (Kalbfleisch and Prentice, 2002) allows to determine medians most correctly and in addition allow to determine the distribution of development times. AFT models were adjusted to the data using the survreg procedure of the survival package in R statistics (Therneau, 2020; Therneau and Grambsch, 2000). For development times, adult longevity and oviposition-time, log-error distributions were assumed; the lognormal, log-logistic, and Weibull model were tested as distribution link function and the most appropriate distribution link function was chosen according to the maximum likelihood. The *ln* median times until occurrence of the events were calculated for each temperature from the intercept and shape parameters of the AFT model and submitted to nonlinear regression analysis (using the nls procedure; R statistics). ILCYM provides several different models that are adequate for describing the relationship between temperature and median development time, mortality, adult senescence and oviposition time, and average fecundity per female. For example, >20 models that might describe temperature-dependent development in insects were available and tested (see the list of models in the user manual for ILCYM 4.0). These functions generally are fitted in term of rates (1/median time); however, in ILCYM and in this study the functions were fitted in terms of *ln*-times. In addition, lower developmental thresholds and the thermal requirements for each life stage were calculated by means of linear regression between temperature and observed development rates (Campbell et al., 1974) using only data points within the linear range (data point at high temperature outside the linear range were deleted). Survivorship in immature life stages was calculated from the relative frequency of surviving test insects. Different nonlinear models (remodeled parabolic functions) available in the ILCYM package were fitted by regression to describe the mortality rate in each life stage and fecundity by temperature. The most appropriate model for describing the temperature effect on any of the above parameters was chosen by comparing corrected Akaike’s Information Criterion (AIC_c_) (Hurvich and Tsai, 1989), which penalizes stronger extra parameters in the model then uncorrected AIC (Akaike, 1973).

After establishing all required temperature-functions for describing the insects’ life history, the “model builder” implemented in the ILCYM software generated an overall temperature-driven phenology model for the species in the R code that can be further deployed in a variety of simulations. For validating the established model, life tables consisting of 100 individuals were simulated stochastically using the respective temperature records measured in the validation experiments (age-stage life tables) that were established at ambient fluctuating temperature. Each simulation was repeated four times. The software uses the shape parameters of the fitted distribution models (AFT models) for development and oviposition time, a random number for mortality in each life stage (individuals for which the random number exceeded the expected temperature-based mortality rate were considered as survivors that developed into the next stage, otherwise they were considered deceased) and sex determination (a random number <0.5 simulated a female and a number >0.5 simulated a male insect), and the standard error of the function fitted to fecundity per female as the stochastic components for simulating the life history of each individual. Simulated and observed life tables were analyzed using standard methods as described in supplementary methods.

Differences in development times, mortality rates, oviposition periods, fecundity per female, and resulting life table parameters—namely the net reproduction rate (*R*_0_), mean generation time (*T*), intrinsic rate of natural increase (*r*_m_), finite rate of increase (*λ*), and doubling time (*Dt*) between simulated and observed life tables were statistically evaluated by using z-scores [eq. 1] and t-statistics

(1) $$z=\frac{observed\;value-simulated\;value}{standard\;deviation\;of\;the\;simulated\;value}$$

Survival and reproduction were much higher in the life tables established at fluctuating temperature, which gave the impression that the vitality of whiteflies is positively affected if temperature fluctuates. To avoid that the final model underestimates the pest risk at fluctuating temperatures we adjusted some parameters of the model functions related to survival and reproduction. The parameters were adjusted using the first life table established at fluctuating temperature in Cusco. The multiplication factors were empirically determined so the life cycle predictions of the model approached as much as possible those obtained under the fluctuating temperature of one location used for calibration and is similar to what is performed in CLIMEX software (CLIMEX v3 user manual. Step 5, page 47, Sutherst et al., 2007). After adjustment, the remaining three life tables established at fluctuating temperature (one from Lima and two from Cusco) were used to validate the modified model.

After model validation, life table parameters for *T. vaporariorum* were simulated from the established model over a range of constant temperatures, as well as over a sinusoidally varying temperature regime of mean temperature ±5 °C for comparison, according to Maia et al. (2000) by using the approximate method (approximate estimate for *T*).

## 3 Results

### 3.1 Development and its distribution

The variation in development times among individuals in the immature life stages across all temperatures was best described by using a log-logistic distribution link function for eggs and nymphs and a Weibull link for puparium in the accelerated failure time (AFT) model (see Table 1). All three link functions revealed highly significant common scale parameters (for each life stage *P* < 0.001), thus adequately describing the variability in development times observed among individuals in each stage. The AFT models revealed a significant effect of temperature on the development times in each life stage (in all stages P < 0.01, see Table 1). Expected median development times resulting from the coefficient of the AFT model decreased significantly with increasing temperature, with the exception that the development time increased again in eggs and puparia at 32 °C and 28 °C, respectively, indicating a delay in development due to high temperature. Because all test insects died at 10° and 32 °C during nymph stage, the development times in nymphs and puparia could not be determined at these temperatures.

**Fig. 1:**
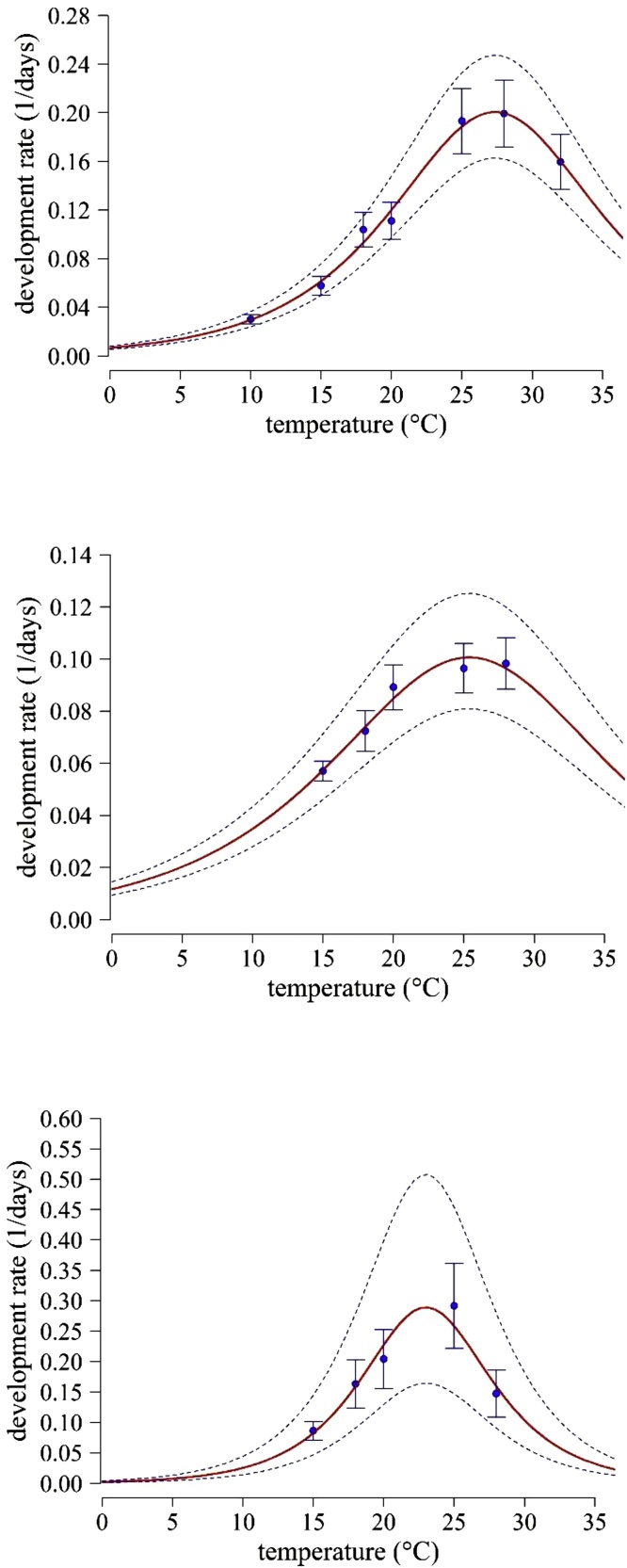
The relationship between temperature and median development rates for immature life stages of *T. vaporariorum* (A: eggs, B: nymph, C: puparium). The models (Janisch model), were fitted in terms of *ln*-development time. Broken lines represent 95 % confidence limits for the fitted model. Markers are observed median development rates (batches). Bars represent 95 % confidence limits of observed data points.

The relationships between temperature and median developmental rates in all three evaluated immature life stages, egg, nymph (N_1_-N_3_), and puparium (N_4_, often referred to as the fourth nymphal instar) were, among several statistically good-fitting models, best described by a Janisch model (Janisch, 1932; Fig. 1, Table 2). The models explained >91 % of the variation in median development times by temperature in each stage (Fig. 1, Table 2). The fastest development, estimated by the parameter *T_opt_* in the Janisch model, was at temperatures of about 27°, 25° and 23 °C for egg, nymph, and puparium stages, respectively.

**Table 2:** Models and estimated parameters fitted to describe temperature-dependent median development rates (1/day) in immature life stages of *T. vaporariorum*.

Life stages	Parameter estimates of the Janisch (1932) model^A^			*F*-value	*df*_1, 2_	*P*	adj. *R*^2^
	*D_min_*	*T_opt_*	*k*				
Egg	4.98 (±0.22)***	27.4 (±0.5)***	0.15 (±0.007)***	249.3	2, 4	<0.001	0.988
Nymph	9.94 (±0.22)**	25.4 (±0.5)**	0.11 (±0.015)*	40.4	2, 2	0.024	0.952
Pupa	3.46 (±0.39)*	23 (±0.37)***	0.24 (±0.028)*	22.5	2, 2	0.043	0.915

Numbers in parenthesis are standard errors. Parameter values significantly different from zero are indicated by asterisks (P < 0.05 = *, P < 0.01 = **, P < 0.001 = ***).

A The equation of the Janisch model (Janisch, 1932) is $r\left(T\right)={\left\{\frac{D_{\min}}{2}\times \left(\exp\left(k\left[T-T_{opt}\right]\right)+\exp\left(-k\times \left[T-T_{opt}\right]\right)\right)\right\}}^{-1}$ where *r*(*T*) is the development rate at temperature *T*, *T_opt_* the temperature at which the development rate is at maximum, and *D_min_*, and *k* are fitted constants.

A linear regression between temperature and development rates, by eliminating the data points at ≥28 °C at which development declined due to high temperature, revealed a lower theoretical temperature threshold for development at 6.9 (SE ±0.7) ºC, 1.4 (±3.8) ºC, and 11 (±0.5) ºC for eggs, nymphs, and puparia, respectively. Based on these thresholds, eggs, nymphs, and puparia require a heat accumulation of 110.8 (±11.4), 230 (±51.5) and 45.5 (±3.4) degree-days (DD), respectively, to complete the life stage.

### 3.2 Immature mortality

**Fig. 2:**
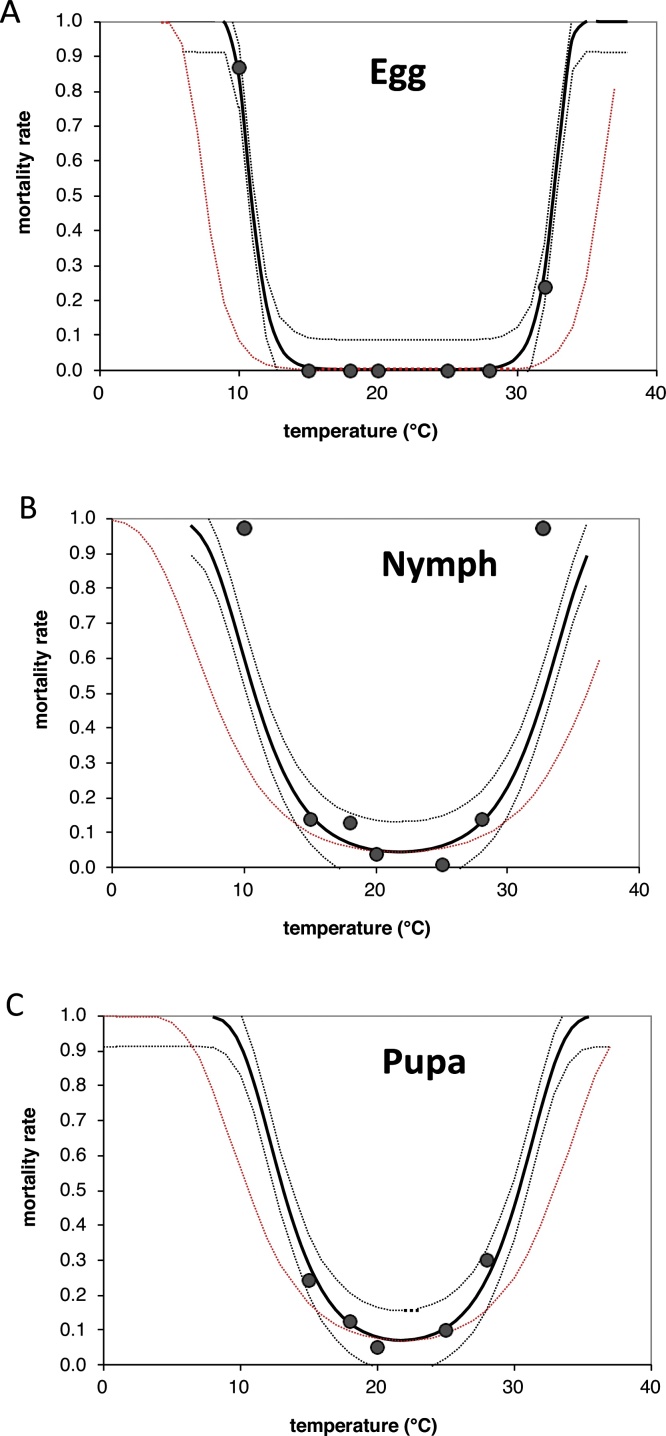
Temperature-dependent mortality ratios of *T. vaporariorum* immature life stages (A: eggs, B: nymph, C: puparium); dots: observed data; lines: nonlinear models fitted; dashed lines: upper and lower 95 % confidence limits of the model. Dashed red lines represent the model used for fluctuating temperature (adjusted model).

The effect of temperature on the mortality of *T. vaporariorum* immature stages (Table 1) was best described by the following nonlinear model (Table 3; Fig. 2), originally developed by the ILCYM team (Sporleder et al., 2017).

(2) $$m_{i}\left(T\right)=\;1-\frac{H_{i}}{\left(1+\text{exp}\left[-\frac{T-T_{opt}}{B_{i}}\right]\right)\times \left(1+\text{exp}\left[-\frac{T_{opt}-T}{B_{i}}\right]\right)}$$

where *m_i_*(*T*) is the overall percent mortality in life stage *i*, *T_opt_* is the temperature (in °C) at which mortality is at minimum, and *H_i_* and *B_i_* are fitted parameters. The model was fitted in different nested forms by setting particular parameters globally for the three life stages. The model with a global parameter for optimum temperature, *T_opt_*, was most appropriate; revealed by AIC_c_ efficiency ratios and by comparing the simpler model (*T_opt_* set global) with the more complex model (individual parameter *T_opt_* for each life stage) using an F-ratio test (*F*-ratio = 3.73, df = 8, 10, P = 0.062). This estimated the optimum temperature for survival in all three life stages to be 21.8 °C (Table 3). The model predicts increasing mortality as temperature deviates from the optimum temperature, indicating limits for survival at around 10° and 30 °C (Fig. 2). The mortality curve after the correction/change of parameter B is indicated by a red dashed line in Fig.2 and extends the temperature limits for survival.

**Table 3:** Estimated parameters of the nonlinear model fitted to describe temperature-dependent mortality in immature life stages of *T. vaporariorum*.

Life Stages	Parameter estimates of the model^A^		
	*T_opt_*	*B_i_*	*H_i_*
Egg	↑	0.903 (±0.999)	3.9 × 10^−6^ (±2.9 × 10^−6^)
Nymph	21.79 (±6.613)**	1.179 (±<0.001)***	6.32 × 10^−6^ (±0.0002)**
Pupae	↓	2.389 (±0.227)***	0.018 (±0.003)***

BNumbers in parenthesis are standard errors. Parameter values significantly different from zero are indicated by asterisks (P < 0.05 = *, P < 0.01 = **, P < 0.001 = ***).

A The models fitted was eq. 2 described in the text using *T*opt as a global parameter (*F* = 156.5, *df* = 6, 12, *P* < 0.001, *R*^2^ = 0.9874).

### 3.3 Adult lifespan and fecundity

**Fig. 3:**
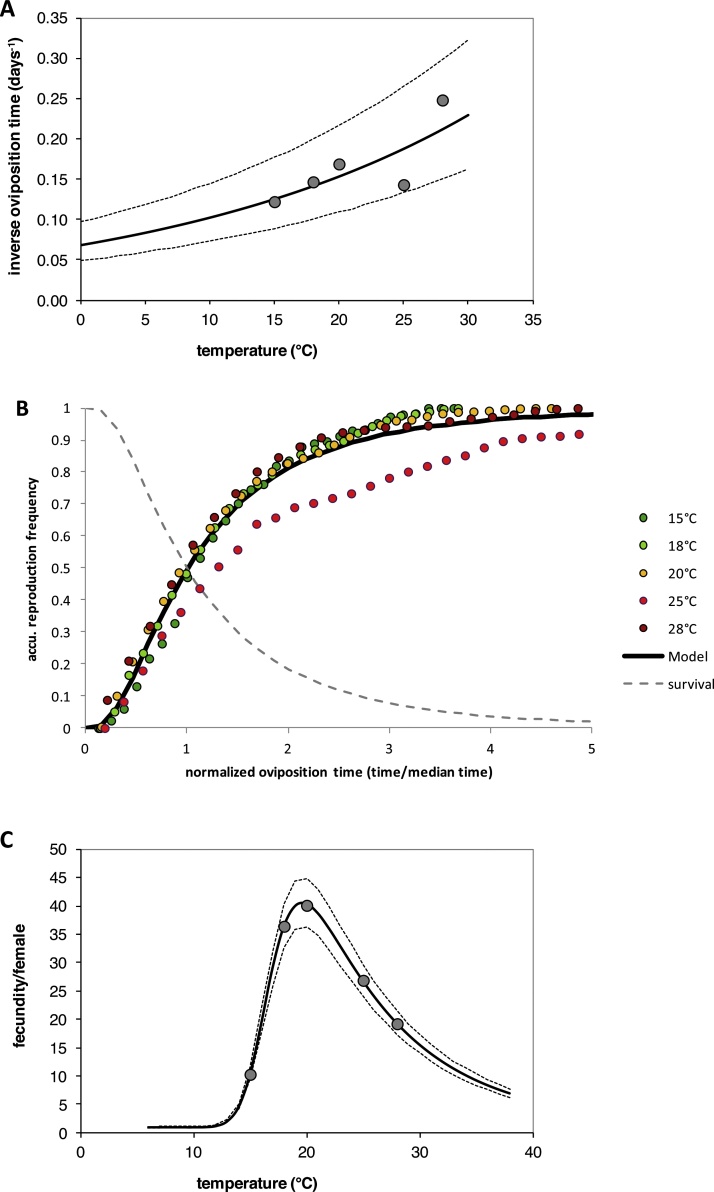
Fecundity of *T. vaporariorum* and its dependence on temperature and female age; (A) temperature-dependent inverse oviposition time (day^−1^) of *T. vaporariorum* females, (B) cumulative proportion of reproduction in relation to normalized female age, and (C) total fecundity per female. In A) and C) dots are observed data; solid lines are fitted models (exponential model in A and a parabolic model (see Table 5) in C); and broken lines are 95 % confidence limits for the fitted model. In B) colored dots are observed data (accumulated mean reproduction/total reproduction per female) at indicated experimental temperatures; bold line is the lognormal distribution model fitted to oviposition data, and the dashed grey line is the expected proportion of surviving females revealed from female survival data (for AFT models, see Table 4).

The variability of adult survival time and median oviposition time was relatively high within each temperature tested (Table 4). The AFT model revealed no significant effect of temperature in the survival time of adults (see Table 4); although the survival times decreased irregularly but gradually with increasing temperature from 7.2 to 4.4 days in females at 15 °C and 28 °C, respectively. The lifespan of males was about 25 % shorter than of females across all temperatures. Median oviposition time decreased gradually from 8.4 days at 15 °C to 4 days at 28 °C (Table 4). The AFT model revealed a significant effect of temperature on the oviposition time; however, significant differences between the groups could only be observed between the lowest (15 °C) and highest (28 °C) temperature tested (Table 4). Variation in survival times of females and males, and oviposition periods among individuals across all temperatures was best described by using a lognormal error distribution (Table 4). The relationships between temperature and survival time of adult *T. vaporariorum* females and males, and oviposition time were best described by simple exponential models (Table 5; Fig. 3a). At some temperatures, median female survival time was shorter than the median oviposition time, which was because a proportion of females died a few days after emergence without laying any eggs (see the relationship between the simulated female survival and normalized oviposition time in Fig. 3b). An AFT model using an additive factor differentiating between female lifespan and oviposition time revealed no significant differences between median female lifetime and median oviposition time (P = 0.858), that means, females reproduced their entire lifetime with almost unchanged daily reproduction rates.

**Table 4:** Median survival times, median oviposition times and total fecundity per female of *T. vaporariorum* adults at different constant temperatures, resulting from AFT modeling.

Temp.	N^A^	Median survival time			Median oviposition time			Mean fecundity		
(°C)	(f / m)	(days)^C^			(days)			(eggs/female) (±STD)	CL(95%)	max.^D^
15	29/40	7.2 (3.3-11)a			8.4 (6.2-10.5)a			10.3 (±13.8)	(5.3-15.3)a	[47]
18	42/35	7.7 (2.6-12.7)a			6.8 (4.9-8.6)ab			36.4 (±32.8)	(26.5-46.4)b	[124]
20	59/35	5.7 (2.1-9.4)a			5.9 (4.3-7.5)ab			40.1 (±53.6)	(26.4-53.8)b	[268]
25	36/57	6.1 (2.3-10)a			7 (5-9.1)ab			26.9 (±42.4)	(13-40.7)ab	[176]
28	42/30	4.4 (1.4-7.5)a			4 (2.8-5.2)b			19.2 (±24.6)	(11.8-26.7)ab	[111]
Model B		lognormal			lognormal					
l_male_^E^=		−0.288 (±0.0974)**								
*ln*(scale)		−0.0757 (±0.0366)*			−0.256 (±0.0094)***					
Scale δ		0.9271 (±0.0712)*			0.7741 (±0.0207)***					
α = 1/ δ		1.08 (±0.08)*			1.29 (±0.03)***					
		Likelihood ratio test			Likelihood ratio test					
		*ln* L	ΔDeviance	*df*	*ln* L	ΔDeviance	*df*	ANOVA^F^		
Intercept only		−1315.3	660.2	150	−18103.3	1393.5	134	*F*_(4, 203)_ =	6.83	
l for each Temp.		−1304.3	638.2	145	−17955.4	1056.7	130		(P<0.001)	
Saturated model		−985.2		(n=152)	−17288.4		(n=136)			
*F*(*df*_x_,*df*_x−1_)		0.9997 (P=0.58)			10.4 (P=0.017)					

A N is the number of females (f) and male (m) adult individuals evaluated at each temperature.

B δ is the scale of the selected distribution link function for survival and oviposition time; the figures in () are SE of *ln*(δ), δ, and α (values significantly different from zero are indicated by asterisks (P < 0.05 = *, P < 0.01 = **, P < 0.001 = ***). The accumulated senescence and oviposition frequency in relation to normalized age (time/median time) is calculated according to the selected distribution link function; for example, for the log-logistic link function: accu. frequency = 1-(1/(1+*x^α^*)), where *x* is the normalized age (determined through rate summation), and *α* = 1./δ.

C Figures are for females. Numbers in parenthesis are 95 % confidence limits based on *t*-distribution (a heterogeneity factor, *H* = *deviance*/*df*, was included to calculated the limits). Medians followed by different letters in the same columns are significantly different (P < 0.05) according to the AFT model.

D Numbers in [] are maximum numbers of eggs/female at each temperature.

E Adult sex was used as an additive factor in the AFT model; according to the parameter, the lifetime of males was 25 % (±5.1 %) shorter than of females.

F ANOVA was performed on *ln*-transformed fecundities (x’= *ln*[x + 1]), where variance between groups were homogeneous (Levene test: P = 0.14).

**Table 5:** Estimated parameters of the non-linear models fitted to describe the relationship between temperature and adult senescence rates, oviposition time^−1^, and average fecundity per females for *T. vaporariorum*.

Response variable	Parameter estimates of the model^A^				*F* value	*df*_1,2_	*P*	R^2^
	Intercept	Slope	additive Factor					
Oviposition time^−1^	0.068 (±0.023)*	0.0405 (±0.0153)*	n.a.		37.5	1, 13	<0.001	0.7428
Female senescence rate	“	“	−0.0106 (±0.0594)^ns^					
Male senescence rate	“	“	−0.3615 (±0.0581)***					
	*Tl*	*Bl*	*Bh*	*H*				
Mean fecundity/female	12.9 (±2.6)***	1.86 (±1.94)^ns^	39.5 (±59.8)^ns^	34.9 (±9.8)***	575.4	1, 3	0.031	0.9994

A The equation for inverse oviposition time, and female and male senescence rate in relation to temperature, *T*, is: $rate=ln\left(\frac{1}{Intercept\times exp\left(Slope\times T\right)}\right)+\;additive\;Factor$ and the equation for fecundity per female relation to temperature, *T*, is:$\text{ln}fecundity=exp\left(\frac{H}{exp\left(\left(1+exp\left(\frac{-\left(T-Tl\right)}{Bl}\right)\right)\times \left(\left(1+exp\left(\frac{-\left(Tl-T\right)}{Bh}\right)\right)\right)\right)}\right)$.

Fecundity per female was extremely variable, ranging from zero to 268 eggs per females observed at 20 °C (see Table 4). The standard deviations appear strongly related to mean fecundity and, as expected, the variance was not equal over the temperatures tested (Levene test: p < 0.001). A Levene test revealed homogeneous variances across all temperatures after *ln*-transformation of the data (*F* = 1.7, *df* = 4, 203, *P* = 0.146), and ANOVA on *ln*-transformed numbers of eggs produced per female revealed a significant effect of temperature on fecundity (*F* = 6.8, *df* = 4, 203, *P* < 0.001). Group-wise comparison indicated significant differences in fertility between 15 °C (lowest reproduction) and 18° or 20 °C (highest reproduction) (Table 4).

The relationship between temperature and mean fecundity per females was significantly best described by a nonlinear parabolic model (see Table 5, Fig. 3c). This model predicts an asymmetric parabolic temperature-dependent reproduction curve with an optimum temperature at ∼20 °C and temperature limits for reproduction at ∼10 °C and >38 °C. The model explained over 99.9 % of the variation in mean fecundity per female by temperature; although the within temperature variation in reproduction per females was extremely high. The proportion of females that died without laying any eggs was quite low at 18 °C (5 %) and 20 °C (8 %) but increase at 25 °C (22 %) and 28 °C (33 %) and was highest at 15 °C (41 %) which largely agrees with optimum and threshold temperature for fecundity predicted by the established model (Fig. 3c). The relationships between temperature and survival time of adult *T. vaporariorum* females and males, and oviposition time was best described by a simple exponential model (Table 5; Fig. 3a). Combining this model with the established distribution link function, the cumulative proportion of egg production in each temperature in relation to normalized female age (expressed as oviposition time/median oviposition time) is demonstrated in Fig. 3b.

### 3.4 Model validation and adjustment to fluctuating temperature

Simulated life table parameters resulting from the model compiled using the functions established from the data collected at constant temperature predicted poorly the life table parameters resulting from the life tables established at fluctuating temperature in Cusco and Lima. The model overestimated mortality rates in immature life stage and underestimated adult survival times and reproduction (Table 6 ). The daily temperature fluctuations measured during the course of life table experiment at fluctuating temperature frequently fell outside the tolerable (permissive) temperature range for whitefly populations predicted by the model based on constant temperatures. For example, minimum and maximum temperatures measured during the course of the first life table experiment in Cusco were 4.6 °C and 35.3 °C, respectively, while average daily minimum and maximum temperature was 8.5 °C and 28 °C, respectively. Whilst the model predicted temperature limits for population development (permissive range) around 14 °C and 32 °C, the insects exposed to fluctuating temperature in Cusco revealed extreme high physiological performance (fast development, high survival rates and high reproduction rate resulting in high population increase rate) and the model clearly overestimated harmful effects due to extreme high and low temperatures, particularly on survival rates and fecundity when used for fluctuating temperature conditions. Therefore, we adjusted the base model for better convergence with the first life table established at fluctuating temperatures in Cusco (see Discussion for rationale behind this) and used the remaining three life tables for validating the modified model. The following modifications were made: a) the parameter *B* in the function for describing temperature-dependent mortality in immature life stages (eq.2) was increase by a factor of 1.3 for extending the temperature limits for survival in all immature life stages; b) survival and oviposition time were increased by a factor of 2, and c) fecundity per female was increased by a factor 4.

**Table 6a:** Comparison of simulated (original model) and observed life history parameters of *T. vaporariorum* obtained for the three life tables established in Cusco and the life table established in Lima.

		Cusco (1^st^ cycle)				Cusco (2^nd^ cycle)				Cusco (3^rd^ cycle)					Lima (1^st^ cycle)			
Avg. daily temp. cycle		8.5°C-28°C				10.1°C-27.7°C				10°C-27.6°C					10°C-27.6°C			
		Sim.		Obs.	P^A^	Sim.		Obs.	P	Sim.		Obs.		P	Sim.		Obs.	P
Life-table parameters																		
*r_m_*	0.021		(±0.009)	0.069	0.002	0.022	(±0.024)	0.0720	0.014	0.031	(±0.026)		0.0772	0.016	0.056	(±0.007)	0.0794	0.005
*R_0_*	2.6		(±0.743)	30.5	0.000	2.25	(±2.167)	43.4	<0.001	2.94	(±0.522)		96.9	<0.001	8.09	(±2.29)	112.9	<0.001
*GRR*^C^	12.31		(±10.73)	64.6	0.025	14.81	(±4.182)	104.5	<0.001	15.06	(±5.43)		212.1	<0.001	19.65	(±3.74)	380.7	<0.001
*T*	37.95		(±1.7)	49.3	0.001	34.86	(±3.6)	52.4	0.002	35.11	(±2.071)		59.3	<0.001	36.74	(±0.83)	59.5	<0.001
*λ*	1.021		(±0.009)	1.072	0.002	1.022	(±0.025)	1.075	0.013	1.031	(±0.027)		1.080	0.015	1.058)	(±0.008	1.083	0.005
*Dt* (days)	33.49		(±19.38)	10.0	0.027	31.65	(±27.08)	9.6	0.078	22.36	(±26.14)		9.0	0.108	12.28	(±1.62)	8.7	0.010

Development time (days)																		
Egg		10.3	(±0.29)	11.5	0.003	9.4	(±0.3)	12.7	<0.001	9.05	(±0.33)		12.4	<0.001	12.14	(±0.31)	13.6	0.002
Nymph		15.13	(±0.96)	19.4	0.003	14.2	(±0.67)	19.2	<0.001	14.24	(±0.6)		21.0	<0.001	13.79	(±054)	18.1	<0.001
Pupa		7.35	(±1.13)	6.0	0.033	7.2	(±0.9)	6.4	0.054	7.6	(±1.25)		7.2	0.38	6.9	(±0.34)	7.4	0.018
Total		32.79	(±1.81)	36.9	0.024	30.8	(±1.34)	38.3	<0.001	30.86	(±1.5)		40.6	<0.001	32.81	(±0.78)	39.1	<0.001

Mortality (%)																		
Egg		0.25	(±0.051)	0.020	0.003	0.15	(±0.086)	0.000	0.014	0.131	(±0.105)		0.020	0.05	0.0	(±0.014)	0.150	<0.001
Nymph		0.24	(±0.045)	0.255	0.202	0.247	(±0.207)	0.140	0.112	0.233	(±0.032)		0.173	0.93	0. 101	(±0.025)	0.176	0.006
Pupa		0.482	(±0.114)	0.014	0.003	0.418	(±0.028)	0.058	<0.001	0.362	(±0.024)		0.000	<0.001	0.191	(±0.133)	0.057	0.051
Total mort.		0.703	(±0.15)	0.28	0.006	0.627	(±0.302)	0.19	0.148	0.574	(±0.157)		0.19	0.014	0.272	(±0.136)	0.33	0.67

Adult survival and fecundity																		
F surv. (days)		8.09	(±2.09)	22.6	<0.001	9.06	(±2.56)	21.4	<0.001	8.13	(±0.88)		43.7	<0.001	8.40	(±1.37)	42.3	<0.001
STD (F surv.)^B^		6.66	(±1.48)	13.1	<0.001	10.43	(±2.06)	13.4	0.148	7.46	(±0.83)		22.8	<0.001	8.89	(±1.62)	30.0	<0.001
Fecundity/f		1.07	(±0.39)	80.3	<0.001	1.77	(±0.48)	92.4	<0.001	1.89	(±0.53)		201.9	<0.001	6.63	(±1.05)	297.2	<0.001
STD (Fecun.)		3.89	(±0.25)	67.8	<0.001	4.80	(±0.49)	78.4	<0.001	5.29	(±0.42)		122.4	<0.001	10.49	(±0.46)	217.8	<0.001
eggs/f/day		1.56	(±0.22)	3.1	<0.001	1.59	(±0.18)	3.9	<0.001	1.64	(±0.18)		4.3	<0.001	2.32	(±0.14)	6.1	<0.001
STD (egg/f/d)		0.69	(±0.02)	1.6	<0.001	0.74	(±0.02)	1.6	<0.001	0.74	(±0.096)		1.5	<0.001	0.90	(±0.05)	2.0	<0.001

A P-values revealing significant differences between observed and simulated values (P < 0.05) are underlined.

B STD is standard deviation.

C Gross Reproduction Rate.

**Fig. 4:**
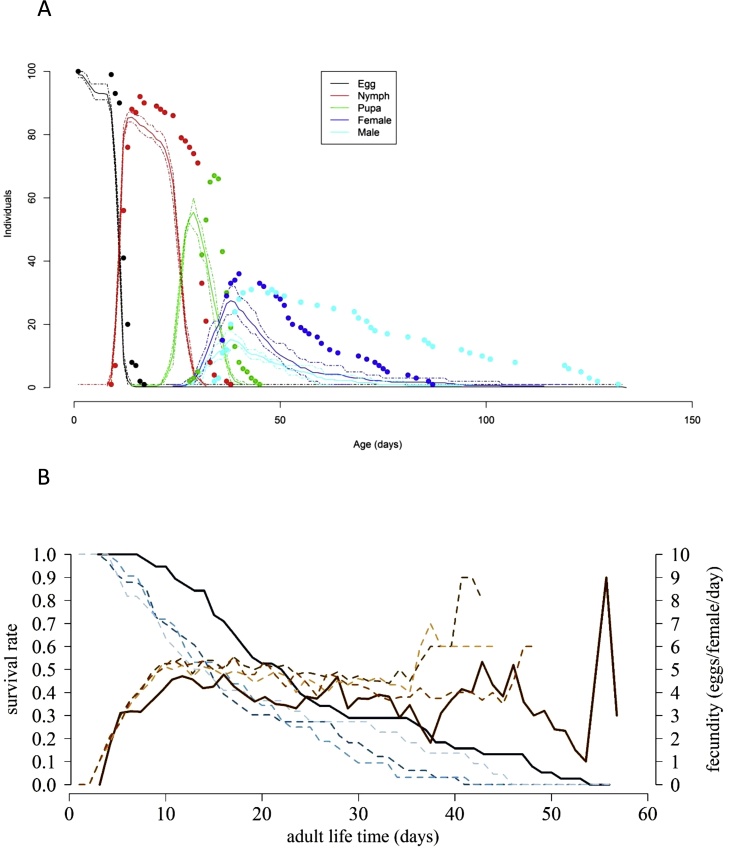
Comparison of life table results obtained for *T. vaporariorum* in Cusco (first life table) with outputs of four stochastically simulated life tables. A) Age-stage specific survival rates; dots are observed data of indicated life stages, lines are stochastic simulation outputs (full line: average of four life table simulations; scattered lines: minimum and maximum values obtained from the four simulations). B) Age-specific survival rates (blue lines) of adult females and fecundity (brown lines; full lines: observed data, scattered lines: results obtained from four stochastically simulated life tables).

Convergence between simulated results using the modified model and observed development times, age-specific survival rates and fecundity observed in the first life table established in Cusco is demonstrated in Fig. 4. Development times in immature life stages were about 11 % underestimated while total immature mortality was about 40 % overestimated (correspondingly, total immature survival was 25 % underestimated) when compared with observed data (Table 6a, b). Female survival time, fecundity and oviposition frequency was quite well predicted. Population parameters were mostly well predicted; the only significant discrepancy was with the mean generation time (Table 6), which was about 5.5 % underestimated. Statistical differences in life history parameters between stochastically simulated and the four observed life tables are shown in Table 6. The z-scores revealed no significant differences in life table parameters between simulated and observed results for the second and third life table established in Cusco. For the life table established in Lima development times were significantly underestimated (−17 %), immature survival overestimated (+23 %), (correspondingly, total immature mortality was −9 % underestimated), and fecundity significantly underestimated (−60 %) (see Table 6). However, simulated and observed life table parameters resulted quite similar. The only significant discrepancy (-42 %) was with the net reproduction rate.

**Table 6b:** Comparison of simulated (adjusted model) and observed life history parameters of *T. vaporariorum* obtained for the three life tables established in Cusco and the life table established in Lima.

		Cusco (1^st^ cycle)				Cusco (2^nd^ cycle)				Cusco (3^rd^ cycle)				Lima (1^st^ cycle)			
Avg. daily temp. cycle		8.5°C-28°C				10.1°C-27.7°C				10°C-27.6°C				10°C-27.6°C			
		Sim.		Obs.	P^A^	Sim.		Obs.	P	Sim.		Obs.	P	Sim.		Obs.	P
Life-table parameters																	
*r_m_*	0.0676		(±0.003)	0.0693	0.534	0.0763	(±0.003)	0.0720	0.145	0.0781	(±0.001)	0.0772	0.387	0.0864	(±0.011)	0.0794	0.537
*R_0_*	23.6		(±3.7)	30.5	0.063	32.6	(±7.4)	43.4	0.143	49.1	(±31.9)	96.9	0.134	64.6	(±4.5)	112.9	<0.001
*GRR*^C^	189		(±69)	64.6	0.072	188	(±58)	104.5	0.151	236	(±23)	212.1	0.288	378	(±27)	380.7	0.909
*T*	46.6		(±1.3)	49.3	0.031	45.6	(±4.6)	52.4	0.137	48.3	(±7.4)	59.3	0.136	48.9	(±6.7)	59.5	0.111
*λ*	1.070		(±0.003)	1.072	0.534	1.079	(±0.003)	1.075	0.145	1.081	(±0.001)	1.080	0.387	1.090	(±0.012)	1.083	0.534
*Dt* (days)	10.3		(±0.4)	10.0	0.533	9.1	(±0.4)	9.6	0.144	8.9	(±0.1)	9.0	0.382	8.1	(±1.2)	8.7	0.634

Development time (days)																	
Egg		10.6	(±0.1)	11.5	<0.001	10.6	(±1.4)	12.7	0.135	10.1	(±1.6)	12.4	0.134	12.3	(±0.1)	13.6	<0.001
Nymph		15.3	(±0.2)	19.4	<0.001	15.4	(±2.5)	19.2	0.134	16.0	(±3.3)	21.0	0.134	14.2	(±0.3)	18.1	<0.001
Pupa		6.7	(±0.4)	6.0	0.031	6.2	(±0.1)	6.4	0.258	7.0	(±0.2)	7.2	0.432	5.9	(±0.2)	7.4	<0.001
Total		32.6	(±0.4)	36.9	<0.001	32.3	(±4)	38.3	0.134	33.1	(±5)	40.6	0.134	32.4	(±0.4)	39.1	<0.001

Mortality (%)																	
Egg		0.099	(±0.023)	0.020	<0.001	0.020	(±0.018)	0.000	0.273	0.013	(±0.005)	0.020	0.134	0.000	(±0)	0.150	–
Nymph		0.187	(±0.032)	0.255	0.033	0.163	(±0.028)	0.140	0.412	0.162	(±0.009)	0.173	0.204	0.073	(±0.021)	0.176	<0.001
Pupa		0.267	(±0.049)	0.014	<0.001	0.191	(±0.092)	0.058	0.149	0.174	(±0.124)	0.000	0.160	0.108	(±0.024)	0.057	0.038
Total mort.		0.463	(±0.04)	0.28	<0.001	0.335	(±0.099)	0.190	0.142	0.317	(±0.091)	0.19	0.161	0.172	(±0.015)	0.33	<0.001

Adult survival and fecundity																	
F surv. (days)		22.3	(±22)	22.6	0.840	21.2	(±1.8)	21.4	0.927	28.1	(±10.5)	43.7	0.135	20.8	(±1.3)	42.3	<0.001
STD (F surv.)^B^		26.1	(±17.9)	13.1	0.428	16.5	(±2.2)	13.4	0.156	21.2	(±3.1)	22.8	0.611	16.3	(±3.3)	30.0	<0.001
Fecundity/f		66.5	(±71.5)	80.3	0.375	79.5	(±11)	92.4	0.240	109.6	(±61.5)	201.9	0.134	120.0	(±7.8)	297.2	<0.001
STD (Fecun.)		67.9	(±55.9)	67.8	0.311	66.0	(±0)	78.4	<0.001	66.0	(±0)	122.4	<0.001	90.4	(±0)	217.8	<0.001
eggs/f/day		2.9	(±3)	3.1	0.686	3.4	(±0.3)	3.9	0.148	3.5	(±0.6)	4.3	0.136	5.0	(±0.1)	6.1	<0.001
STD (egg/f/d)		0.8	(±0.7)	1.6	<0.001	1.1	(±0.4)	1.6	0.151	1.0	(±0.4)	1.5	0.140	1.7	(±0.1)	2.0	<0.001

A P-values revealing significant differences between observed and simulated values (P < 0.05) are underlined.

B STD is standard deviation.

C Gross Reproduction Rate.

### 3.5 Life table parameters

**Fig. 5:**
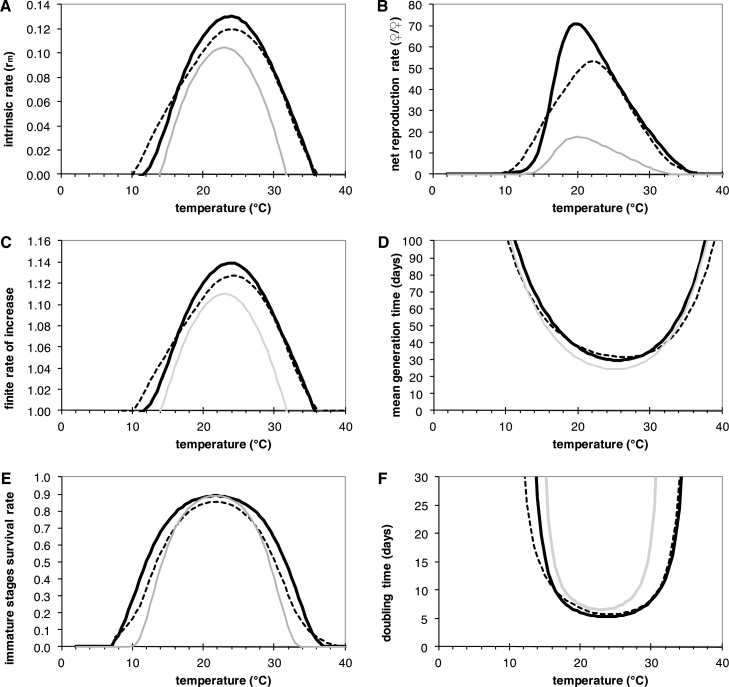
Life table parameters of *T. vaporariorum* simulated using the adjusted phenology model developed in this study over a temperature range from 0 to 40 °C. (A) intrinsic rate of natural increase (*r_m_*), (B) net reproduction rate (*R*_0_) [females/female], (C) finite rate of increase (λ), (D) mean generation time (T) [days], (E) Immature stages survival rate, (*S*) doubling time (*Dt*) [days]. Black line: adjusted model prediction if temperature is held constant; scattered black line: adjusted model prediction if temperature fluctuates ±5 °C (x-value ±5 °C); grey line: original model prediction at constant temperatures.

The modified model predicts *T. vaporariorum* population development at constant temperature within the range of 11.5 °C–35.5 °C (Fig. 5). Maximum population growth is expected at around 24 °C with a finite rate of increase, *λ*, of 1.137 (1.14) (see Fig. 5c), which corresponds with a population doubling time of 5 days. In contrast, the original model established from the constant temperature experiments (thermal performance curves for the original model are visualized as a grey line in Fig. 5) predicted much narrower limits for population growth at 14 °C and 32 °C. Thermal performance curves at fluctuating temperatures generally flatten as explained by the Kaufmann effect (Worner, 1992). For illustrating the Kaufmann effect anticipated by the modified model, life tables were simulated using a daily temperature cycle of ±5 °C. These simulations were carried out in hourly intervals using a sine wave function between the daily minimum temperature (average temperature minus 5 °C) and maximum temperature (average temperature plus 5 °C). These temperature fluctuations flatten the performance curves (see scattered lines in Fig. 5), with population development thresholds at minimum mean temperature of 10 (±5) °C and maximum mean temperature of 36.5 (±5) °C.

## 4 Discussion

The greenhouse whitefly, an important pest on numerous vegetable crops, is a vector of several important viral diseases which are largely managed by control of the insect itself. Pest management strategies should ideally be based on understanding pest ecology in agroecosystems. Assessing the pest’s population growth potential is an important aspect that can be determined through the study of life table parameters and insect phenology. Several phenological models have been developed for insect pests that can predict life parameters under a temperature regime, taking into account the total life cycle of insects (Sporleder et al., 2004; Ngowi et al., 2017). Comprehensive research has been conducted on studying the effect of temperature on *T.vaporariorum* in different crops (Table 7). Examples about using this information for developing temperature-based models for the pest include simple degree-days (DD) models for predicting development times (see references mentioned in Table 7) and more advanced models (Yano, 1988; van Roermund and van Lenteren, 1992v) that focuses on describing population dynamics, e.g. of the tritrophic system host plant (tomato)-insect-parasitoid (Encarsia). In this study we provide experimental data showing how life table parameters of the greenhouse whitefly are affected by temperature and generate a model that can simulate the full life history of this species in potato.

**Table 7:** Longevity, fecundity, oviposition frequency, oviposition time, develop time, survival rate and intrinsic rate of increase (*r_m_*) of *T. vaporariorum* reported from other studies and compared to simulation results based on our model.

				Longevity (days)				Fecundity (eggs/f)		Oviposition frequency (eggs/f/d)		Oviposition time		Mean development time (days)		Survival rate (%)		*r_m_*	
Autor	Cultivar	Temp. (°C)		Rep.*		Sim.		Rep.	Sim.	Rep.	Sim.	Rep.	Sim.	Rep.	Sim.	Rep.	Sim.	Rep.	Sim.
Manzano and Lenteren, 2009	Beans: Chocho		19		22.6		11.5	32.6	159.7	1.4	3.5			29.3	27.2	97.4	99.6	0.04	0.095
	Beans: Chocho		22		17.5		10.2	33.3	143.8	1.9	3.6			24.7	20.9	81	99.7	0.06	0.116
	Beans: Chocho		26		5.9		8.7	8.6	96	1.4	2.8			20.3	19.5	52.3	99.4	0.04	0.112
	Beans:ICA-Pijao		19		5.5		11.5	127	159.7	3.6	3.5			30.4	27.2	74	99.6	0.07	0.095
	Soybean 'Cristalina'	23±3		27.6		9.6		337	127.6			25.6	38.2	15.5	19.6	72.6	99.7	0.16	0.119
	IAC - Carioca Pyatã'	23±3		22.75		“		102.2	“			26.6	“	16.5	“	74.5	“	0.14	“
	'IAPAR - 57′	23±3		22.5		“		172.4	“			23.3	“	16	“	63.5	“	0.16	“
	'Jalo Precoce'	23±3		17.65		“		70.6	“			15	“	16.4	“	81	“	0.12	“
	'IAC - Bico de Ouro’	23±3		25.1		“		199.4	“			21.3	“	15	“	84.6	“	0.16	“
	'IAC - Maravilha'	23±3		26.4		“		238	“			26	“	15.5	“	60.3	“	0.12	“
Prijović et al., 2013	Tomato:Narvik	27±2		18.57		8.3								21.8	20.3				
	Tomato: NS-6	27±2		20.33		“								21.4	20.3				
	Tomato: Tamaris	27±2		24.1		“								22.1	20.3				
	Tomato: Alliance	27±2		25.17		“								21.8	20.3				
	Tomato: Marko	27±2		27.2		“								20.6	20.3				
Burnett, 1949	7	15		50.5		13.13		93.6	61.7	1.9	1.2				42				
Hussey & Gurney, 1957	Tomato	15.6		31.3		13.13		131.5	61.7	4.2	1.2		52.6						
Lloyd, 1922	Tomato	17.3		34		12.27		92	126.4	2.7	2.6		49						
van Es, 1982	Tomato: Dombo	22.5		57.4		9.95		215.6	137.7	3.7	3.5		39.5					0.07	0.12
	Tomato: Portanto	22.5		68.6		9.95		219	“	3.2	“		“					0.07	“
	Tomato: Moneydor	22.5		37.1		9.95		90.4	“	2.7	“		“					0.06	“
González-Dufau et al., 2018	Tomato	20.8		12.29		10.7		218	156.4	2.2	3.4		42.4	33.1	22.8	63	99.7	0.07	0.11
	Potato	20.8		4.79		“		27.6	“	5	3.7		“	41.8	“	13	“	0.06	“
Tsueda and Tsuchida, 1998	Tomato	20				11			161.2		3.7		43.8	31.8	24.5	66.7	99.7		0.10
	Tomato	30				7.4			62.2		2.16		28.9	26.11	26.3	23.1	96.7		0.08

Rep.: Reported, Sim.: Simulated.

To compare our model predictions with observed data reported by other authors (Table 7), predictions were made using averages temperatures reported. Based on this, our model over, or underestimated the development time by 1–10 days (0.1–18 %) except the case with González-Dufau et al. (2018) where our model underestimated by 11–19 days (30–45 %). In the other variables such as adult longevity, fertility and oviposition time our model underestimated reported values in most cases. Our model overestimated survival rates in all cases, however, the temperatures reported were all quite close to the optimal temperature for survival according to our model. Several authors estimated the intrinsic rate of population increase, *r*_m_, based on their data (Table 7). The model predictions of this life table parameter overestimated the results reported from all studies, except for Campos et al. (2003). Ideally, we would have made predictions with fluctuating temperatures, however, these were not reported in such detail and actual temperature fluctuations could have influenced the simulation results. Other factors that might have caused divergences between reported and simulated life history parameters could be due to different *T. vaporiarorum* biotypes or haplotypes (Barboza et al., 2019; Wainaina et al., 2018), cultivar resistance or simply a result of the apparently inherent high variability in life table parameters found in the species. Nevertheless, the established functions adequately predicted the result in most of the previous studies and therefore could be helpful in modeling development times of the whitefly in other host crops and regions.

The lower theoretical temperature threshold for development found in this study also is within the range of other studies (Ortega and Carapia, 2020). Weber (1931) found a lower threshold temperature for development of eggs and the first three larval instars of 8 °C on tobacco and for L4 larvae a few degrees lower. Other authors such as Osborne (1982) estimated a lower threshold temperature of 8.3 °C by linear regression using data of Stenseth (1971), whereas Madueke and Coaker (1984), using their own data estimated the threshold temperature in the range between 7–11.5 °C. However, degree-day models interpolate the linear relation between environmental temperature and insect development rate observed between the highest and lowest tolerable temperature in which development is usually linear. This is the range in which higher temperatures promote higher rates of development, although some non-optimal temperatures below or above certain temperature generally cause either a retardation or acceleration of development rates, respectively (Scotta, 2013). The difference between development predicted by linear models, like a DD model, and the development expected under variable temperature conditions by using nonlinear models is called the “Kaufmann effect” or the “rate summation effect” (Worner, 1992). Therefore, the nonlinear model presented in this paper is more recommendable than the degree day model because it predicts more reasonable development when temperature falls outside the optimal temperature during daily temperature fluctuations. How the simulated life table parameters adjust in regard to the Kaufmann effect is demonstrated in Fig. 5.

The model predicts increasing mortality of immature stages as temperatures deviates from the optimum temperature, indicating limits for survival between around 10° and 30 °C, with higher mortality rates during nymph and puparium stages (Fig. 2) and is consistent to other authors (Evert and van Schutte, 1983; Weber, 1931; Yano, 1981; Scotta, 2013). However, previous authors have noted that high mortality was only observed when temperature was constantly high, and at fluctuating temperatures with short peaks of 30 °C or more, increased mortality was not observed (Yano, 1988; Vianen et al., 1987; Lenteren et al., 1989), indicating that at high temperatures the duration of exposure is important (Scotta, 2013). Indeed, van Roermund and van Lenteren (1992) observed that immature mortality was not very high in greenhouses that reached temperatures exceeding 30 °C, but that these peak temperatures did not exceed more than 5 h in duration. This observation likely also explains why our original model significantly overestimated mortality rates in immature life stages as compared to life tables determined under extreme fluctuating temperatures (reaching as high as 30–35 °C in Cusco; Table 6). This ecological behavior is similar to that reported by Scotta, 2013, where during autumn there were days of maximum temperatures above 36 °C and minimum temperatures below 10 °C, and the thermal sum appeared to have been overestimated.

The temperature effects on *T. vaporariorum* adult survival time observed in this study generally also follow the same trend of that reported by several authors, whom reported a negative effect on whitefly survival and adult longevity as temperature increased (Manzano and Lenteren, 2009; van Roermund and van Lenteren, 1992v; Table 7). Our data reveal a significant effect of temperature on adult survival time (AFT model); however, the within each temperature variation in adults survival time was extremely high (within group variability was higher than the variability due to temperature). In previous literature the survival time is most often reported to be around 30 days (Scotta, 2013), but according to our data whiteflies can survive also much longer (under fluctuating conditions >100 days).

The initial model developed based on life tables determined at constant temperatures, however, poorly predicted those determined in studies at naturally fluctuating temperatures, principally due to an overestimation of immature mortality rates and an underestimation of adult survival and reproduction rate. We have not observed such substantial differences between laboratory and field collected data in previous studies (Aregbesola et al., 2020; Sporleder et al., 2004, 2016; Mujica et al., 2017). This gives the impression that the species *T. vaporariorum* is a special case showing high variability in adult survival and reproduction in response to variable temperature. Length of exposure to extreme temperatures influences the detrimental effect of extreme temperatures and may have led to over- and under-estimation of mortality and survival rates respectively (Scotta, 2013), whereas the exposure to constant temperatures and light regimes might have provided a general stress factor for the insects detrimentally affecting their life parameters overall (Colinet et al., 2015). In addition, fluctuating temperatures have been reported to increase, decrease, or have no effect on the adult lifespan and reproduction of an insect due to various reasons (reviewed by Colinet et al., 2015). Since the female survival time and reproduction capacity observed, by using females from the same original colony, in our experiments performed higher at fluctuating temperature experiments, it is justified, in view for not underestimating the risk of population establishment by using the model, to adjust survival time and reproduction too. By including correction factors for different functions (extending the temperature range for immature survival, and increasing the survival time and reproduction of females) based on the first life tables established at fluctuating temperatures we could achieve convergence of the predicted and observed results (Fig. 4a). Simulated and observed life table parameters based on the other three life tables established at fluctuating temperature subsequently resulted quite similar. Nevertheless, it would be important to confirm these correction factors by determining life tables at fluctuating temperatures similar to those observed in the field but under controlled conditions to disentangle them from other factors that may also influence life history.

Other complementary factors like host plant, geographic whitefly strain, humidity and light intensities can influence the whiteflies’ life history (van Roermund and van Lenteren, 1992v). For example, a large effect of different hosts on greenhouse whitefly reproduction and population growth potential can readily be observed from comparing the differences in *r_m_* (intrinsic rate of natural increase) values estimated for *T. vaporariorum* on different host plants (see Table 7). There are few data available concerning the effects of potato as a host on the reproductive capacity of *T. vaporariorum* at similar conditions as tested here (e.g., González-Dufau et al., 2018), making any specific comparisons difficult. Few experiments have been performed to study other factors, such as light intensity, air humidity or whitefly density. Weber (1931) studied the effect of humidity on immature mortality and found lowest mortality at 70–80 % RH. He also measured oviposition frequency in the dark, which was low compared to the oviposition frequency at daylight conditions. Hussey and Gurney (1958) on the other hand did not find differences in oviposition at different light intensities or daylengths. In a broader sense, incorporating other factors known to influence *T. vaporariorum* dynamics into the model such as weather-influenced reproductive and dispersal behavior of the pest, host plant diversity, natural enemy dynamics, relative humidity, and rainfall could enhance the accuracy of predictions in the future.

Our original model predicted temperature limits for populations beyond development between 14 °C and 32 °C. After including the correction factors for fluctuating temperatures, the model indicated that growth and reproduction of *T. vaporariorum* could be sustained at constant temperature between 11.5 °C and 35.5 °C, while at fluctuating temperature the thresholds for minimum and maximum mean temperature extend to about 10 °C and 36.5 °C, respectively, due to the Kaufmann effect (Fig. 5). Maximum number of eggs and female offspring were produced at 20 °C, but shifted towards 25 °C under fluctuating temperatures, with maximum population growth at around 24 °C with a finite rate of increase, *λ*, of 1.137 and a population doubling time of 5 days. Finite rate of population growth and doubling time are the most important parameters describing population increase. These results agree with previous reports of Weber (1931) who found a lower and upper threshold temperature for oviposition of 10 °C and 37 °C on tobacco. González-Dufau et al. (2018), also reported maximum oviposition by *T. vaporariorum* at 20.8 °C in tomato, with the highest intrinsic rate of natural increase occurring 20.8 °C.

In conclusion, the final modified phenology model and simulated life-table parameters for *T. vaporariorum* reflect the temperature-dependent population growth potential of the insect and could quite well explain its development in contrasting environments in Peru. The model produced reasonable life-table parameters for the whitefly based on temperature and could therefore, if linked to Geographic Information Systems, produce maps that allow predictions of population and distribution changes in response to changing temperatures as influenced by global warming. Thus, this model could be used for geography specific risk assessment of the whitefly as well as diseases it transmits and is presented in an accompanying paper (Gamarra et al., 2020 accompanying paper) in this issue considering PYVV.

## CRediT authorship contribution statement

**Heidy Gamarra:** Data curation, Investigation, Project administration, Supervision, Writing - original draft, Writing - review & editing. **Marc Sporleder:** Formal analysis, Methodology, Validation, Supervision, Writing - original draft, Writing - review & editing. **Pablo Carhuapoma:** Data curation, Formal analysis, Software, Visualization. **Jürgen Kroschel:** Funding acquisition, Resources. **Jan Kreuze:** Conceptualization, Funding acquisition, Methodology, Project administration, Writing - original draft, Writing - review & editing.
