# Supplementary material for: A temperature-dependent phenology model for the greenhouse whitefly Trialeurodes vaporariorum (Hemiptera: Aleyrodidae)
Source: Virus Res. 2020 Nov;289:198107. doi: 10.1016/j.virusres.2020.198107 (PMC7569604; doi:10.1016/j.virusres.2020.198107)
Supplement: Supplementary file 1 [file mmc1.docx]

**Supplementary methods**

Age-stage structured life tables of daily development, survival, and reproduction of each individual, were analyzed according to the age-stage, two-sex life table theory as described by Chi (Chi and Liu, 1985; Chi and Su, 2006). The population parameters calculated were age-stage specific survival rate (*S_xj_*: the probability that a newly laid egg will survive to age *x* and stage *j*), age-stage specific fecundity (*f_xj_*: the mean fecundity of females at age *x*), age-specific survival rate (*l*_x_: the probability that a newly laid egg survives to age *x*), and age-specific fecundity (*m*_x_: the mean fecundity of individuals at age *x*).

In the age-stage, two-sex life table, *l*_x_ and *m*_x_ are calculated as (Chi and Liu, 1985)

$$l_{x}=\sum_{j=1}^{k} s_{xj}$$

$$m_{x}=\frac{\sum_{j=1}^{k} s_{xj}f_{xj}}{\sum_{j=1}^{k} s_{xj}}$$

where *k* is the last stage of the study cohort.

The net reproductive rate (*R*_0_) represents the total number of offspring that can be produced by an individual female during its lifetime and is calculated as:

$$R_{0}=\sum_{x=0}^{\infty} l_{x}m_{x}$$

The gross reproduction rate (GRR) represents the average number of female offspring by a female if the female survived throughout the whole oviposition period and is calculated as:

$$GRR=\sum_{x=0}^{n} m_{x}$$

The intrinsic rate of increase (*r*) is estimated by solving the Euler-equation using the iterative bisection method (with age indexed from zero) as follows:

$$\sum_{x=0}^{\infty} e^{-r\left( x-1 \right)}l_{x}m_{x}=1$$

The finite rate of increase (*λ*) is calculated as follows:

$$\lambda=e^{r}$$

The mean generation time (*T*) is defined as the length of time that a population requires to increase to the *R*_0_-fold of its size at the stable age-stage distribution (the value corresponds is the average time between two consecutive generations and matches almost with the time span until 50% of the egg of the following generation are laid). The parameter is calculated as follows:

$$T=\frac{lnR_{0}}{r}$$

Age-stage life expectancy (*e_xj_*), i.e. the time that an individual of age *x* and stage *j* is expected to live, was calculated according to the method described by Chi and Su (Chi and Su, 2006) as:

$$e_{xj}=\sum_{i=x}^{n} \sum_{j=y}^{m} {s'}_{xj}$$

where *n* is the number of age groups and *m* is the number of stages, and *s*′*_ij_* is the probability that an individual of age *x* and stage *j* will survive to age *i* and stage *y*. The age-stage reproductive value (*v_xj_*) was defined as the contribution of individuals of age *x* and stage *j* to the future population (Yang et al., 2015). In the age- stage, two-sex life table, it is calculated as:

$$v_{xj}=\frac{e^{-r\left( x-1 \right)}}{s_{xj}}\sum_{i=x}^{n} e^{-r\left( x-1 \right)}\sum_{j=y}^{m} {s'}_{xj}f_{xj}$$

The survival rate, *s_xj_*, to each age-stage unit is calculated as:

$$s_{xj}=\frac{n_{xj}}{{ns}_{0,1}}$$

where *n_0,1_* is the number of eggs used at the beginning of life table study and *n_xj_* is the number of insects that survived to age *x* and stage *j*. Because the total number of eggs (*E*_x_) laid by all female adults was recorded (the fourth life stage) at age *x*, female age-specific fecundity *f_x,_*_4_ is calculated as:

$$f_{x,4}=\frac{E_{x}}{n_{x,4}}$$

and the net reproductive rate is calculated as:

$$R_{0}=\sum_{x=0}^{\infty} \sum_{j=1}^{m} s_{xj}f_{xj}$$

where *m* is the number of life stages. The age-specific survival rate (*l_x_*), the age-specific fecundity (*m_x_*), the finite rate of increase (λ), the intrinsic rate of increase (r), and the mean generation time (T) were calculated as previously described for the individually reared method (fluctuating temperature).

For group reared method (constant temperature experiments), the age-specific life expectancy (*e_x_*), i.e. the time that individuals of age *x* are expected to live, was calculated as:

$$e_{x}=\frac{\sum_{i=x}^{n} l_{i}}{l_{x}}$$

where *l_i_* is the probability that an individual of age 0 will survive to age *i*. The reproductive value (*v_x_*) as the contribution of individuals of age *x* to future population, is calculated as:

$$v_{x}=\frac{e^{-r\left( x-1 \right)}}{l_{x}}\sum_{i=x}^{n} e^{-r\left( x-1 \right)}l_{i}m_{i}$$

References

Chi, H., Liu, H., 1985. Two new methods for the study of insect population ecology. Bull. Inst. Zool. Academia Sinica (24), 225–240.

Chi, H., Su, H.-Y., 2006. Age-stage, two-sex life tables of *Aphidius gifuensis* (Ashmead) (Hymenoptera Braconidae) and its host *Myzus persicae* (Sulzer) (Homoptera: Aphididae) with mathematical proof of the relationship between female fecundity and the net reproductive rate: Braconidae) and Its Host *Myzus persicae* (Sulzer) (Homoptera: Aphididae) with Mathematical Proof of the Relationship Between Female Fecundity and the Net Reproductive Rate. Environ Entomol 35 (1), 10–21.

Yang, Y., Li, W., Xie, W., Wu, Q., Xu, B., Wang, S., Li, C., Zhang, Y., 2015. Development of *Bradysia odoriphaga* (Diptera: Sciaridae) as affected by humidity: an age–stage, two-sex, life-table study. Appl Entomol Zool 50 (1), 3–10.
